# Sex‐Specific Associations Between Systemic Inflammation and Brain Health in Aging: Evidence from a Multi‐Ethnic Canadian Cohort

**DOI:** 10.1002/alz70861_108892

**Published:** 2025-12-23

**Authors:** Katie L Vandeloo, Rachel Yep, Jordan A Chad, Tulip Marawi, Alexander J Nyman, Madeline Wood Alexander, Georgia Gopinath, Angelina Zhang, Simran Malhotra, Rohina Kumar, Sarah‐Mei Chen, Silina Z Boshmaf, Harleen Rai, Walter Swardfager, Sandra E. Black, Maged Goubran, Jennifer S Rabin

**Affiliations:** ^1^ Hurvitz Brain Sciences Program, Sunnybrook Research Institute, Toronto, ON Canada; ^2^ Rotman Research Institute, Baycrest Health Sciences., Toronto, ON Canada

## Abstract

**Background:**

Females tend to exhibit greater reductions in brain volume and cognition with age compared to males. Furthermore, females are twice as likely as males to be diagnosed with Alzheimer’s disease, and often experience a more severe disease course. Emerging evidence suggests that inflammation resulting from estradiol depletion during the menopause transition may play a key role in these age‐related sex disparities. However, research examining sex‐specific inflammatory pathways and their role in dementia remain limited. This study aimed to address this gap by examining whether sex moderates the influence of inflammation on brain and cognitive outcomes in a multi‐ethnic Canadian cohort.

**Method:**

Cross‐sectional data were collected from N=137 (mean age=65.8±6.7, 97 females (71%)) cognitively unimpaired older adults participating in the Canadian Multiethnic Research on Aging (CAMERA) study at Sunnybrook Research Institute in Toronto, Canada. Inflammation was measured with plasma‐derived C‐reactive protein (CRP). Markers of brain health included whole‐brain gray matter volume (GMV) derived from FreeSurfer (v.8), and whole‐atlas fractional anisotropy (FA) derived from UKF tractography‐based white matter analysis. Cognition was measured with experimental and neuropsychological tests, including domain‐specific scores for processing speed, executive function, and episodic memory. Linear regression analyses examined whether sex moderated the effect of inflammation on brain and cognitive outcomes. Covariates included age and years of education, as well as total intracranial volume for GMV analysis, and total white matter hyperintensity volume for FA analysis.

**Result:**

CRP levels were higher in females than males, after adjusting for age and years of education (partial *f* ^2^= 0.06, pFDR=0.03). Sex significantly moderated the association between CRP and brain health outcomes. Specifically, higher CRP levels were more strongly associated with lower GMV (partial *f* ^2^=0.12, pFDR<0.001) and with lower FA (partial *f* ^2^=0.06, pFDR=0.01) in females than males. Sex did not moderate the relationship between CRP and cognition (pFDR>0.05).

**Conclusion:**

Inflammation may have a stronger negative impact on gray matter volume and white matter microstructural integrity in females compared to males, potentially contributing to sex differences in neurodegeneration and dementia risk. Future research should investigate the underlying biological mechanisms driving these associations to inform the development of sex‐specific interventions targeting inflammation‐related neurodegenerative processes.

